# Neurons from skin mimic brain holes

**DOI:** 10.18632/oncotarget.13709

**Published:** 2016-11-29

**Authors:** Ilya Bezprozvanny, Sergey L. Kiselev

**Affiliations:** Federal Clinical Research Center for Physical-Chemical Medicine FMBA, Moscow, Russia; Vavilov Institute of General Genetics RAS, Moscow, Russia

**Keywords:** huntington, reprogramming, calcium, antisense, therapy, Neuroscience

Huntington’s disease (HD) is a neurodegenerative disorder characterized by motor, behavioral and cognitive abnormalities that worsen until death. HD is caused by expansion of a CAG repeat tract of the *huntingtin* gene, encoding mutant Huntingtin (mHtt) protein with an expanded polyglutamine (polyQ) tract (> 39 Qs). The pathological hallmark of HD is the loss of medium spiny neurons (MSNs) in the striatum, which accounts for the major clinical symptoms of the disease [1]. The early symptoms of HD precede neuronal loss, suggesting an abnormal function of striatal neurons in early stage HD. HD management is limited to symptomatic treatments and supportive care. Transgenic animal models that have generated most of the research that influences the field today carry hundred and more CAG repeats. HD mouse models carry fragment or full length human Htt gene on artificial chromosomes or contain CAG repeat expansion in the context of the mouse Htt gene. In addition to rodent models, *C. elegans*, *D. Melanogaster*, sheep, and monkey transgenic HD models were generated [2]. Studies with these transgenic models provided valuable insights into HD pathogenesis. However, behavioral and cellular changes observed in animals are sometimes very different from those observed in the human condition and therapies validated in animal models are often not effective in clinical trials.

Extensive analysis of HD brain postmortem samples has been performed [1], but it is difficult to make conclusions about cause-and-effect and disease initiation from these results. Studies with human cell lines originally obtained from tumor samples and transfected with mutant Htt gene were performed, but these cells carry mutations involved in cell proliferation, apoptosis, and immortality, all of which complicate neurodegeneration pathways analysis. Investigation of live human HD neurons until recently were not feasible, but this limitation has been overcome by development of cell reprogramming technology that makes possible to convert patients fibroblasts obtained from skin biopsies into an immature, embryonic-like cells known as the induced pluripotent stem (iPS) cells [3]. These iPS cells can then be induced to become any cells that mimic the disease process [4].

**Figure 1:**
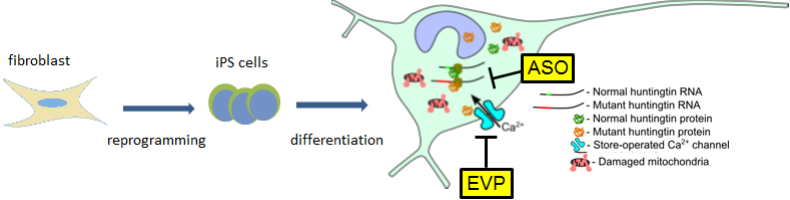
Skin fibroblasts were reprogrammed to pluripotent stem cells that were directed into neurons Disease hallmarks were detected very early during neurons maturation. Antisense oligonucleotides effectively blocked mutant *htt* RNA, while EVP4593 normalized calcium entry.

Multiple studies have been published that describe properties of HD iPS cells [5]. Recently Nekrasov and colleagues have utilized iPS-derived human HD MSN neurons for a series of mechanistic studies [6]. iPS cells were established from patient’s biopsies with most frequently observed moderate number of CAG repeats (40-44). iPS cells generated from mutant fibroblasts were indistinguishable from the wild type however MSNs differentiated from mutant cells readily demonstrated hallmarks of the disease. Impairment of autophagosome/ lysosome system, mitochondria impairment, nuclear indentations were observed in all independently generated patient’s neurons (Figure 1). Neuronal differentiation in vitro usually takes 3-6 months and generates fetal neurons, thus it was not surprising that despite these alterations HD MSNs did not show an increased cell death. Misfolded, damaged, aggregated or unnecessary proteins are degraded by the proteasome. Proteasome functionality declines during the aging process and triggers the onset of age-related diseases. Thus inhibition of proteasome system should mimic cellular aging. Indeed, HD MSNs died twice faster than normal MSNs in response to inhibition of proteasome [6].

One of the pathways implicated in HD pathogenesis is dysregulated calcium (Ca2+) signaling due to direct interaction of mutant Htt with inositol 1,4,5-trisphosphate receptor, an intracellular calcium release channel. Conceptually, the calcium hypothesis links relatively early and minor defects in calcium homeostasis with long-term effects of this dysregulation on brain cells, eventually determining age-associated neurodegeneration. It was demonstrated earlier that store operated Ca_2+_ pathway (SOC) is upregulated in primary MSNs from YAC128 transgenic HD mice and in human neuroblastoma cells transfected with Htt-138Q [7]. However it was unclear whether calcium dysregulation occurs in human cells before the disease onset. Using neurons reprogrammed from skin fibroblasts Nekrasov and colleagues for the first time demonstrated that SOC entry is upregulated in HD human MSNs with a moderate number of polyQ repeats [6].

Since HD is caused by a dominant mutation in either of an individual’s two copies of the *htt* gene, silencing of the mutant RNA will ultimately eliminate disease phenotype in HD neurons. Antisense oligonucleotides therapy targeting Htt gene is in the clinical development now, but this approach eliminates both normal and mutated allele of *htt*. Nekrasov at al demonstrated that specific oligonucleotides complementary to the CAG repeat completely abolished mutant protein synthesis in neurons leaving normal allele expressed. HD neurons cured in such way acquired normal nuclear morphology and normalized calcium homeostasis.

Small molecules offer a great advantage of brain-blood barrier penetration although do not eliminate the cause of the disease. Such molecules can be used to delay onset of these disease or slow down disease progression. Calcium dyshomeostasis is an important factor in the etiology of HD. Small molecule EVP4593 has been shown to inhibit SOC entry in HD mouse primary MSNs [7] and to rescue synaptic spine loss in HD MSN primary cultures and in vivo in HD mice [8]. Remarkably the same molecule completely restored calcium entry and normalized mitochondrial function in human mutant HD neurons established from several individuals, therefore representing a pharmacological agent for potential HD therapeutics in humans. Most strikingly EVP4593 rescued HD neurons from premature cell death in dose dependent manner, implicating an important role of SOC dysregulation in development of cellular disease phenotype [6]. It is worth to mention that reprogramming makes an adult cell go developmentally ‘backwards’ and establish its embryonic potential to develop into different types of tissue cells such as brain, heart, liver, kidney, etc. Therefore, the same iPS cells could be used to differentiate them into heart or liver and to test individual drug toxicity even before going to phase 1 of clinical trial.

An approach using iPS-derived human neurons utilized by Nekrasov at al [6] provided critical insights into cell death pathways in HD neurons and offered additional support to the calcium hypothesis of HD. In the future this platform can be used to facilitate HD drug discovery efforts.
